# *QuickStats:* Rates* of Deaths Attributed to Unintentional Injury from Fire or Flames,**^†^** by Age Group and Urbanization Level**^§^** — National Vital Statistics System, United States, 2018

**DOI:** 10.15585/mmwr.mm6934a8

**Published:** 2020-08-28

**Figure:**
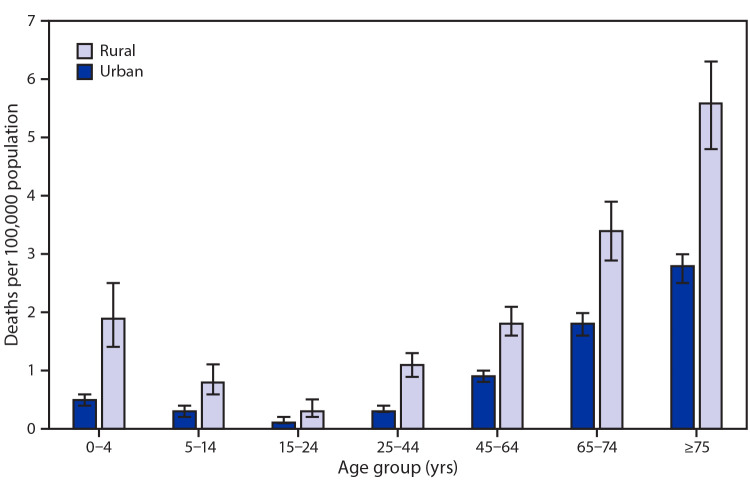
In 2018, the death rates attributed to unintentional injury from fire or flames were lowest among those aged 15–24 years and highest among those aged ≥75 years. In rural areas, death rates decreased with age from 2.0 per 100,000 for persons aged 0–4 years to 0.3 for those aged 15–24 years, and then increased with age to 5.6 for those aged ≥75 years. The pattern was similar for urban areas, where rates were 0.5 per 100,000 for persons aged 0–4 years, decreased to 0.1 for those aged 15–24 years, and then increased with age to 2.8 for those aged ≥75 years. Across all age groups, death rates were approximately two to four times higher in rural areas compared with urban areas.

**Source:** National Center for Health Statistics, National Vital Statistics System, mortality data; 2018. https://www.cdc.gov/nchs/nvss/deaths.htm.

